# Incremental peritoneal dialysis: a 10 year single-centre experience

**DOI:** 10.1007/s40620-016-0344-z

**Published:** 2016-08-31

**Authors:** Massimo Sandrini, Valerio Vizzardi, Francesca Valerio, Sara Ravera, Luigi Manili, Roberto Zubani, Bernardo J. A. Lucca, Giovanni Cancarini

**Affiliations:** 1 O.U. of Nephrology, A.S.S.T. Spedali Civili di Brescia, Piazzale Spedali Civili, 1, 25123 Brescia, Italy; 2 Università di Brescia, Brescia, Italy

**Keywords:** Incremental dialysis, Peritoneal dialysis, Dialysis adequacy, Residual renal function

## Abstract

**Introduction:**

Incremental dialysis consists in prescribing a dialysis dose aimed towards maintaining total solute clearance (renal + dialysis) near the targets set by guidelines. Incremental peritoneal dialysis (incrPD) is defined as one or two dwell-times per day on CAPD, whereas standard peritoneal dialysis (stPD) consists in three-four dwell-times per day.

**Patients and methods:**

Single-centre cohort study. Enrollement period: January 2002–December 2007; end of follow up (FU): December 2012. Inclusion criteria: incident patients with FU ≥6 months, initial residual renal function (RRF) 3–10 ml/min/1.73 sqm BSA, renal indication for PD.

**Results:**

Median incrPD duration was 17 months (I–III Q: 10; 30). There were no statistically significant differences between 29 patients on incrPD and 76 on stPD regarding: clinical, demographic and anthropometric characteristics at the beginning of treatment, adequacy indices, peritonitis-free survival (peritonitis incidence: 1/135 months-patients in incrPD vs. 1/52 months-patients in stPD) and patient survival. During the first 6 months, RRF remained stable in incrPD (6.20 ± 2.02 vs. 6.08 ± 1.47 ml/min/1.73 sqm BSA; p = 0.792) whereas it decreased in stPD (4.48 ± 2.12 vs. 5.61 ± 1.49; p < 0.001). Patient survival was affected negatively by ischemic cardiopathy (HR: 4.269; p < 0.001), peripheral and cerebral vascular disease (H2.842; p = 0.006) and cirrhosis (2.982; p = 0.032) and positively by urine output (0.392; p = 0.034). Hospitalization rates were significantly lower in incrPD (p = 0.021). Eight of 29 incrPD patients were transplanted before reaching full dose treatment.

**Conclusions:**

IncrPD is a safe modality to start PD; compared to stPD, it shows similar survival rates, significantly less hospitalization, a trend towards lower peritonitis incidence and slower reduction of renal function.

## Introduction

Incremental dialysis consists in prescribing a dialysis dose aimed at maintaining total solute clearance near the targets set by guidelines. In peritoneal dialysis (PD), the total amount of blood purification is equivalent to the sum of residual renal function plus the peritoneal dialysis dose [1, 2]. This makes it possible to start dialysis at less than full dose when there is still a significant renal function; afterwards, the dialysis dose is gradually increased to compensate renal function decline and to meet adequacy targets [3, 4].

The incremental approach to peritoneal dialysis (incrPD) was first developed in the late 90s. At that time and for many of the following years, however, incrPD was sometimes mistakenly presented as a way to start dialysis earlier [3–8]. Moreover, shifting consensus regarding the timing of dialysis initiation has increased confusion between “incremental” and “early” dialysis [9, 10]. In 2006, the Kidney Disease Outcomes Quality Initiative (KDOQI) guidelines suggested that nephrologists should evaluate the benefits, risks, and disadvantages of beginning kidney replacement therapy when glomerular filtration rate (GFR) is <15 ml/min [11]; in 2013, dialysis initiation was recommended whenever the patient becomes symptomatic (independently of GFR) [12]; in 2014, instead, it was advised to start dialysis when GFR is <15 ml/min/1.73 m^2^ body surface area (BSA) and/or the patient becomes symptomatic, but, in any case, before GFR falls to 6 ml/min/1.73 m^2^ BSA [13].

A comparison between the results of incrPD as opposed to standard PD (stPD) (full dose) must be done using similar GFR values and excluding cases with an early start of dialysis or with an extra-renal indication for dialysis (i.e. heart failure and end-stage liver disease). The first clinical experiences with incrPD date back to 1999–2002 [7, 8, 14, 15]; initial prescription consisted of 1–2 dwell-times per day. In 2003, incremental automated peritoneal dialysis (APD) was suggested; it consisted of a full daily dose of APD, but only for 3–4 nights per week [16].

Based on these reports, in 2002 we started an incrPD program. This paper analyzes our clinical experience and attempts to provide further information regarding this new approach to the start of dialysis.

## Patients and methods

This cohort study attempts to define the results of the incremental PD approach. All patients who started PD from January 1st 2002 to December 31st 2007 in our center were included. End of follow-up was December 31st 2012, or when the patient stopped PD because of death, shift to hemodialysis (HD), renal transplantation or recovery of renal function.

Residual renal function (RRF) was measured as the mean of creatinine renal clearance and urea renal clearance. Inclusion criteria were as follows: follow-up lasting at least 6 months, RRF at start of PD >3 ml/min/1.73 m^2^ BSA and <10 ml/min/1.73 m^2^ BSA and a “renal indication” for PD. Standard dialysis dose (stPD) was defined as 3–5 dwell-times per day, 7 days a week, for continuous ambulatory peritoneal dialysis (CAPD) and nightly dialysis sessions, seven nights a week, for APD. Incremental dialysis dose (incrPD) was defined as one or two dwell times per day on CAPD.

The eligible patients were divided into two groups according to the dialysis dose with which they started: stPD or incrPD. Choice of PD modality, either CAPD or APD, was made according to patient preference following adequate information and exclusion of clinical contraindications. IncrPD was suggested only to patients who had chosen CAPD, because at that time incremental APD had not been defined yet.

All demographic and clinical data were prospectively recorded in a database (File Maker®, File Maker Inc., Santa Clara, CA, USA). The following baseline data were exported anonymously to an Excel (®Microsoft, Redmond, WA, USA) file: age, sex, primary renal disease, comorbidities, weight and body mass index, RRF and class of peritoneal permeability according to a modified 3.86 % peritoneal equilibration test (PET) done about 3 months after the beginning of PD. Other data exported were: time on PD, adequacy parameters, incidence of peritonitis, hospitalization rate and outcome. RRF was measured monthly in incrPD patients and quarterly in stPD patients. The comparison of adequacy values between incrPD and stPD was made at baseline, after 6 months and at the end of PD or when transition from incrPD to stPD took place.

### Statistical analysis

Continuous data are expressed as mean ± standard deviation (SD) or median and interquartile range (I–III Q) according to their distribution. Comparisons were performed using Student’s *t* test or Wilcoxon test as appropriate. Dichotomous variables were analyzed using the Chi square test. A p value <0.05 was considered as statistically significant. Comparison of survival was done by Kaplan–Meier curves and log-rank test. Multivariate analysis was done by Cox proportional hazards model. The statistical program used was SPSS® version 19 (SPSS Inc., Chicago, IL, USA).

## Results

### Baseline data

The total number of incident patients in PD in 2002–2007 was 178, of whom 39 had a follow-up <6 months, 28 had a RRF <3 ml/min, at start, 5 a non-renal indication for PD, and 1 patient was lost to follow-up. As a consequence, the eligible patients were 105: 42 (40 %) were women and 63 (60 %) men; 29 (28 %) were in the incrPD group and 76 (72 %) in the stPD group; 57 (75 %) patients of the stPD group were on APD and 19 (25 %) were on CAPD. Main baseline data of the two groups are reported in Table 1: no statistically significant difference was found regarding age, gender, weight, body mass index, comorbidity, primary renal disease, RRF or class of peritoneal permeability.

**Table 1:** Baseline data of the two groups: incrPD and stPD

	incrPD	stPD	*p*
Number of patients	29	76	
Male gender	13 (55 %)	50 (66 %)	0.611
Age (years)	63 ± 12	59 ± 18	0.200
Weight (Kg)	63.4 ± 10.2	62.8 ± 16.7	0.837
BMI (Kg/m^2^)	24.3 ± 3.9	23.3 ± 3.7	0.130
RRF (ml/min/1.73 m^2^ BSA)	5.74 ± 1.34	5.42 ± 1.75	0.381
D/P creatinine 4th hour	0.63 ± 0.14	0.62 ± 0.11	0.426

*incrPD* incremental peritoneal dialysis, *stPD* standard peritoneal dialysis, *BMI* body mass index, *RRF* residual renal function, *BSA* body surface area, *D*/*P* dialysate/plasma

### Change of PD modality

Median duration of incrPD was 17 months (I–III Q: 10; 30); 21 incrPD patients shifted to stPD after a median of 17 months (I–III Q: 10; 27). Causes of transition were a reduction in RRF in 19 patients and abdominal hernia in two. Eight patients stopped PD: 5 due to renal transplantation and 1 due to recovery of renal function; 2 patients died. Median duration of stPD was 36 months (I–III Q: 18; 55); 5 patients shifted from CAPD to APD, two because of personal choice, two due to abdominal hernias and one because of inadequate dialysis. A total of 69 stPD patients stopped PD: 27 (39 %) due to renal transplantation, one (1 %) recovered renal function, 10 (14 %) shifted to HD and 32 (46 %) died.

### Adequacy data

A comparison of dialysis adequacy data is shown in Table 2: total (renal + peritoneal) wKt/V (twKt/V) and wClCr (twClCr) at the beginning, after 6 months and at the end of the treatment. Student’s *t* test for paired samples was carried out only between initial data and the 6th month, since at the end of treatment the follow-up periods were dramatically different among patients. At the sixth month Kt/V and wCrCl were significantly lower in stPD (p = 0.012 and 0.004, respectively), but stable in incrPD (p = 0.672 and 0.485). The changes were associated with a significant reduction of the renal contribution to both the urea and creatinine clearance in stPD (p < 0.001 for both), whereas changes were not statistically significant in incrPD.

Changes in peritoneal clearances occurring in stPD mainly depended on changes in the prescribed dose of dialysis.

**Table 2:** Results of renal and peritoneal clearances of creatinine and urea in incrPD and stPD groups

	Initial data			6th month			End of treatment^a^			p value 6th month vs. initial data	
	incrPD	stPD	*p*	incrPD	stPD	*p*	incrPD	stPD	*p*	incrPD	stPD
Number of patients	29	75		25	66		25	65			
twKt/V	2.08 ± 0.38	2.40 ± 0.58	**0.008**	2.13 ± 0.45	2.20 ± 0.43	*0.527*	1.77 ± 0.50	2.01 ± 0.35	0.007	*0.672*	**0.012**
twCrCl (l/w/1.73 m^2^)	81 ± 15	85 ± 18	*0.341*	83 ± 19	77 ± 20	*0.192*	66 ± 27	62 ± 21	*0.460*	*0.485*	**0.004**
Residual renal creatinine clearance	7.55 ± 1.94	7.36 ± 2.21	*0.690*	7.74 ± 2.97	5.81 ± 2.72	**0.004**	5.39 ± 3.85	2.62 ± 3.35	**0.001**	*0.797*	<**0.001**
Peritoneal creatinine clearance	1.98 ± 0.70	2.80 ± 0.93	<**0.001**	2.02 ± 0.63	3.22 ± 1.21	<**0.001**	2.16 ± 0.68	4.08 ± 1.37	<**0.001**	*0.297*	<**0.001**
Renal + peritoneal creatinine clearance	9.53 ± 1.99	10.16 ± 2.52	*0.231*	9.77 ± 2.87	9.04 ± 2.63	*0.255*	7.55 ± 3.66	6.71 ± 2.82	*0.246*	*0.600*	**0.015**
Peritoneal contribution to total creatinine clearance (%)	21 ± 7	28 ± 8	<**0.001**	22 ± 10	38 ± 19	<**0.001**	32 ± 13	70 ± 30	<**0.001**	*0.448*	<**0.001**
Residual urea renal clearance	4.55 ± 1.23	3.81 ± 1.35	**0.014**	4.63 ± 1.42	3.12 ± 1.67	<**0.001**	3.27 ± 2.09	1.4 ± 1.85	<**0.001**	*0.778*	<**0.001**
Peritoneal urea clearance	2.44 ± 0.78	4.50 ± 1.38	<**0.001**	2.54 ± 0.79	4.68 ± 1.18	<**0.001**	2.66 ± 0.81	5.66 ± 1.52	<**0.001**	*0.393*	*0.155*
Renal + peritoneal urea clearance	6.99 ± 1.15	8.30 ± 1.93	<**0.001**	7.17 ± 1.32	7.80 ± 1.54	*0.073*	5.94 ± 1.80	7.06 ± 1.42	*0.246*	*0.490*	**0.018**
Peritoneal contribution to total urea clearance (%)	35 ± 11	54 ± 11	<**0.001**	36 ± 11	61 ± 16	<**0.001**	47 ± 16	82 ± 21	<**0.001**	*0.757*	<**0.001**
Creatinine renal clearance/urea renal clearance	1.70 ± 0.43	2.08 ± 0.92	**0.007**	1.69 ± 0.55	2.16 ± 1.24	**0.007**	1.65 ± 0.33	2.17 ± 1.58	*0.051*	*0.678*	*0.080*
Residual renal function	6.08 ± 1.47	5.61 ± 1.49	*0.160*	6.20 ± 2.02	4.48 ± 2.12	<**0.001**	4.36 ± 2.96	2.03 ± 2.55	< 0.001	*0.792*	<**0.001**

All values except Kt/V are normalized to 1.73 m^2^ of body surface area

Statistically significant values are in bold

Statistically non-significant values are in italic

*twKt*/*V* total weekly Kt/V, *twCrCl* total weekly creatinine clearance

^a^Last value before changing from incrPD to stPD or stopping PD

### Incidence of peritonitis

During a total follow-up of 4603 patient-months, 91 episodes of peritonitis occurred, with an incidence of 1/51 patient-months. When considering only the time spent on the first modality, incrPD or stPD, the incidence of peritonitis was 1/135 patient-months in the incrPD group vs. 1/52 patient-months in the stPD group (Table 3). If all periods at risk are taken into consideration, the incidence of peritonitis in the stPD group becomes 1/46 patient-months (Table 3). In the stPD group, the 19 patients on CAPD had a follow-up of 665 patient-months with an incidence of peritonitis of 1/39, while the 57 on APD had an incidence of peritonitis of 1/58 over a follow-up period of 2267 patient-months. The incidence of peritonitis was 1/39 on CAPD vs. 1/49 on APD when all periods at risk were taken into consideration. Even though the absolute incidence was quite different, the cumulative probability of being peritonitis-free (log-rank test on Kaplan–Meier curves) showed no significant difference between incrPD and stPD or between CAPD and APD.

**Table 3:** Prevalence of peritonitis in the two groups of patients: incrPD and stPD

Only first treatment	Number of patients	29	76
	Follow-up (patient-months)	677	2932
	Number of peritonitis episodes	5	56
	Peritonitis incidence (episode/patient-months)	1/135	1/52
Overall	Number of patients	29	110
	Follow-up (patient-months)	677	3926
	Number of peritonitis episodes	5	86
	Peritonitis incidence (episode/patient-months)	1/135	1/46

Overall: calculated over the entire follow-up period according to the modality in use at that time and considering the patient who changes modality as a new patient

### Hospitalization

**Fig. 1:**
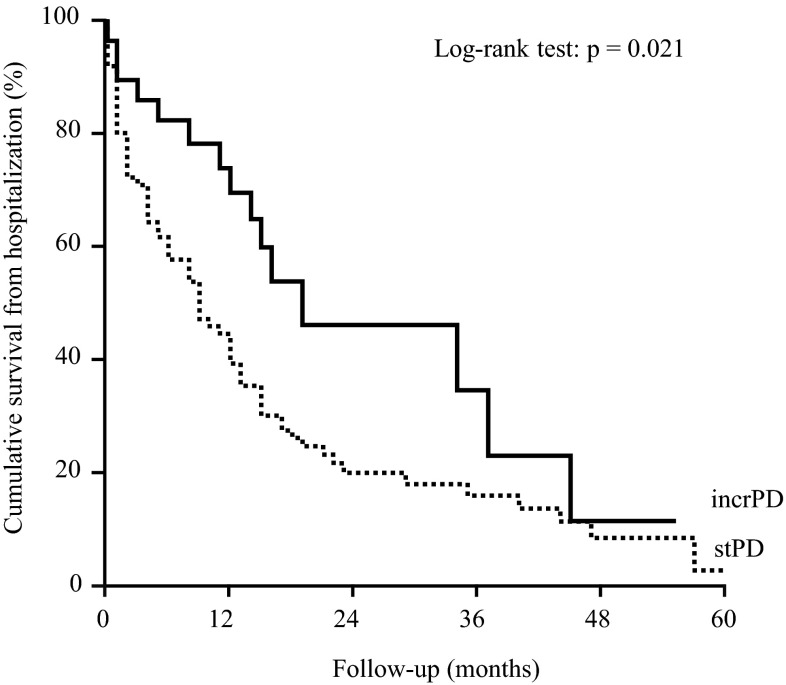
Cumulative probability to be hospitalization-free in incrPD and stPD

Among the patients on incrPD, 30 admissions occurred in 677 patient-months (1/23 patient-months) for a total of 322 days of hospitalization (5.7 days/patient-years). Among the patients on stPD there were 325 admissions in 2932 patient-months (1/9 patient-months) and 3784 days of hospitalization (15.5 days/patient-years). At the “as treated” analysis, the cumulative probability to be hospitalization-free was higher in the incrPD group than in the stPD group (p = 0.021) (Fig. 1); only 20 % of the stPD patients were hospitalization-free after 24 months vs. 45 % of the incrPD group.

### Patient survival

**Fig. 2:**
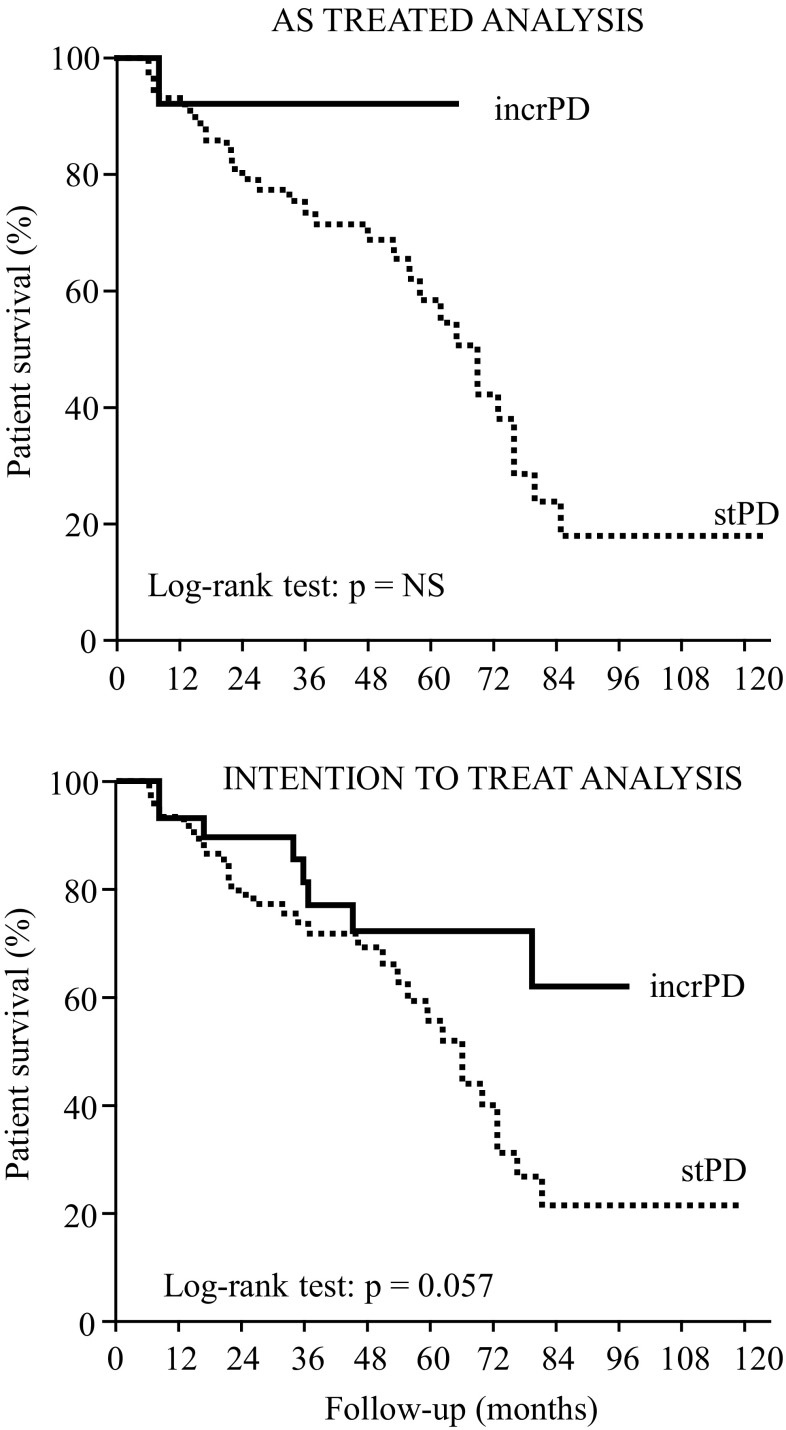
Cumulative probability to survive in incrPD and stPD

At the end of the follow-up, 41 patients (40 %) had died. In the incrPD group, 2 patients died of peritonitis, 1 died due to sepsis/severe infection, 2 due to ischemic heart disease, 1 due to a stroke, 2 due to cachexia and 1 died due to hemorrhagic shock. In the stPD group, 3 patients died of peritonitis, 12 patients died of sepsis/severe infections, 3 from ischemic heart disease, 1 from stroke, 4 due to cachexia, 1 of malignancy, 6 due to other causes and in two cases the cause of death was unknown. Patient survival was not significantly different between incrPD and stPD, according to both the “as treated” and the “intention to treat” analysis (Fig. 2). The Cox hazards regression model was applied to identify those factors (PD modality, age, sex, comorbidity, RRF and urine output) significantly affecting patient survival. Ischemic cardiopathy, peripheral and cerebral vascular disease and cirrhosis (the latter only with “as treated” analysis) were detrimental factors. On the contrary, urine output significantly improved survival (Table 4). PD modality did not affect survival.

**Table 4:** Results of the Cox hazard regressions on patient survival

Method	Factor	HR		95 % CI	p
As treated	Ischemic heart disease	4.269		1.174–7.124	<0.001
	Peripheral/cerebral vascular disease	2.842		1.630–9.330	0.006
	Urine output (l/day)	0.392		0.164–0.934	0.034
	Cirrhosis	2.982		1.037–6.0257	0.032
Intention to treat	Ischemic heart disease	3.297	1.716–6.332		<0.001
	Peripheral/cerebral vascular disease	3.354	1.715–6.555		<0.001
	Urine output (l/day)	0.387	0.181–0.826		0.014

*HR* hazard ratio, *CI* confidence interval

## Discussion

The basic assumption of incremental dialysis is to reach the minimal targets for adequate dialysis by summing renal function and dialysis dose. Mehrotra et al. and Golper first described an early and incremental approach to peritoneal dialysis [3, 4]. Some clinical experiences in incrPD were reported in the 90s; the main features of those studies are summarized in Table 5. The aforementioned studies enrolled only a few patients, without a control group and without any statistical comparison. De Vecchi et al. first reported working activity, degree of rehabilitation and quality of life in incrPD patients; quality of life and social rehabilitation were better preserved with incrPD than with stPD [15].

**Table 5:** Papers published regarding clinical experiences in incrPD

Author, year	Period of time	Study design	No. pts on incrPD	incrPD schedule	Initial GFR (ml/min)	Time on incrPD (patient-months)	Peritonitis rate (episode/patient-months)	Results/outcomes
Williams, 1999 [14]	NA	Pilot study	15	CAPD 1 dwell/day	9.8 ± 1.9	90	1/30	Adequacy (good)
								Hospitalization (3 admissions)
								Survival (patients and technique)
De Vecchi et al., 2000 [15]	1995–1999	Pilot prospective, not controlled	25	CAPD 1–2 dwells/day	6–10	262	1/21	Good rehabilitation with incrPD
								Better quality of life with incrPD
								Adequacy (good)
								Exit-site infections (8 episodes)
								Complications
								Hospitalization (3 days/year)
								Survival (patients and technique)
Burkart et al., 2000 [7]	1997–1999	Non randomized, prospective	13	CAPD 1–3 dwells/day	6.7 ± 2.4	159	1/53	Adequacy (good)
								Complications
								Survival (patients and technique)
Foggensteiner et al., 2002 [8]	1997–2000	Pilot, not randomized, prospective	39	CAPD 1 dwell/day	10	422	1/30	Adequacy (good)
								Complications
								Hospitalization (3.6 days/year)
								Survival (patients and technique)
Neri et al., 2003 [16]	2000–2001	Preliminary experience	5	APD 3–4 sessions/week	7–9	84	none	Adequacy
								Peritonitis
								Compliance
								Complications
								Survival (patients and technique)
Viglino et al., 2008 [27]	2004–2007	Retrospective	11	CAPD 2 dwells/day	7.3 ± 2.7	106	NA	Choice of dialysis modality
								RRF and adequacy (good)
								Technique survival
Domenici et al., 2011 [28]	2000–2008	Retrospective	17	?	6.9 ± 1.1	480	1/48	Reduced rate of loss of RRF
Jeloka et al., 2013 [29]	2006–2011	Retrospective	13	CAPD 1 dwell/day	7.8 ± 2.6	244	1/56	Adequacy (good)
Barràs Sans et al., 2016 [30]	2003–2012	Retrospective	46	CAPD 3 dwells/day	8.0 ± 3.2	1035	1/99	Reduced rate of loss of RRF
								Reduced dose of erythropoietin

*APD* automated peritoneal dialysis, *CAPD* continuous ambulatory peritoneal dialysis. For other abbreviations, see previous tables

As of today, there are no papers which delineate the features, efficacy, feasibility and safety of incrPD. Despite these considerations, incrPD is used more and more often: in Italy, 54 % of PD centers use incrPD and 29 % of patients start PD with the incremental approach [17]. The hypothetical benefits of incrPD may explain its widespread use: better quality of life, reduced glucose exposition, better peritonitis-free survival, longer preservation of residual renal function, cost reduction and increased PD penetration; it is also considered as an ideal bridge to renal transplantation.

Our experience with incrPD began in the late 90s and is based on two assumptions: (a) advanced renal failure and renal replacement therapy are a continuum and should be treated as such [18]; (b) in accordance with the studies regarding peritoneal dialysis adequacy [2], total blood purification is considered as the sum of renal and peritoneal clearance, even though they have different blood purification profiles. Furthermore, we have to clarify the enrolment modality: the choice of PD modality between CAPD and APD, as well as between incrPD and stPD, was up to the informed patient (in the absence of clinical contraindications). The incremental approach was suggested only to patients who had chosen CAPD because, at that time, incrAPD had not been recommended in the clinical literature. This study’s comparison of incrPD vs. stPD was made only in those patients who had started dialysis before 2008 as, after the end of 2007, the positive clinical results yielded by incrPD urged us to suggest it to all patients with RRF ≥3 ml/min/1.73 m^2^ BSA. Because of this, a stPD group for comparison was not available any more.

For a comparison between incrPD and stPD to be made, it is necessary that: groups of patients are comparable, the start of dialysis occurs at similar values of RRF and patients who started PD due to non-renal indication are excluded. The two groups of our study were comparable (Table 1). RRF at the beginning of PD, calculated as the mean of measured creatinine and urea clearance, was in line with the guidelines in force at the time of the study [19]. RRF was not different between the incrPD group and the stPD group, so incrPD was not started at an earlier stage than stPD. It should be noted that to start with incrPD it is necessary to have a good pre-dialysis program and a timely initiation of dialysis; several studies have shown that a good pre-dialysis education program increases the prevalence of patients opting for self-care dialysis [19–25]. As far as adequacy is concerned, patients in both the incrPD group and the stPD group were always above the minimal targets of adequacy [2] even though, as expected, adequacy values were higher on stPD at the beginning of treatment (Table 2).

It is interesting to note that total Kt/V and wCrCl in the incrPD groups remained stable over the first 6 months, whereas they decreased significantly in the stPD group. This could be due to a better preservation of residual renal function in incrPD both for creatinine and urea whose ratios did not significantly change. It should also be noted that the peritoneal contribution to the total clearance is always higher for urea than for creatinine, due to differences in the renal handling and peritoneal permeability of those molecules. The results of this study suggest a protective role of incrPD on RRF which was stable in incrPD in the first 6 months whereas it significantly decreased in stPD (Table 2). This stability could be the reason for a median duration of incrPD of 17 months, which could positively affect the patients’ quality of life on PD due to a lesser burden of dialysis procedures. On this ground it is also clear that patients on incrPD need a closer clinical follow-up to reduce the risk of under-dialysis; in our center, RRF was measured monthly and total clearances quarterly in incrPD.

Peritonitis is a major complication of PD and remains an important cause of drop-out. According to the recommendations of the International Society for Peritoneal Dialysis, the peritonitis rate should be lower than one episode every 18 months. In the literature regarding incrPD, peritonitis rates range from zero (follow-up: 84 patient-months) to one episode/21 patient-months (Table 5). De Vecchi et al. reported that the risk of peritonitis was associated to the number of exchanges [15]. In our study the peritonitis rate was 1/135 patient-months in the incrPD group and 1/52 patient-months in the stPD group (Table 3). The reduced number of connections and the “dry” period could have played a role in reducing the peritonitis rate in incrPD even though it was not statistically significant according to the Kaplan–Meier curves. On the other hand, the low frequency of exchanges of APD, in incrPD, could reduce patient experience, increase the risk of errors and, consequently, the occurrence of peritonitis. According to our results, however, this effect does not appear to be so important. Hospitalization-free survival was significantly better in the incrPD group than in the stPD group; however, it must be considered that the treatment of our patients suffering from peritonitis is done on an inpatient basis. We did not find any papers comparing patient survival in incrPD vs. stPD. In our study, incrPD showed a trend towards better survival at the “intention to treat” analysis, but it was marginally non-significant (Fig. 2). The Cox analysis did not indicate incrPD as a risk factor for mortality (Table 4). The data we collected do not suggest a clear survival benefit with incrPD, but at least they support a non-inferiority of incrPD vs. stPD.

The main biases of our study are: (1) it is a retrospective, one-center study, and (2) the patients were not randomized. As a consequence, the results cannot be generalized. However, its results could be of some help in designing future multicenter randomized studies.

## Conclusions

Incremental peritoneal dialysis, which must not to be confused with an early start of dialysis, provides adequate dialytic doses, has a reduced hospitalization rate compared to stPD, yields a similar patient survival to that observed with stPD, and is time saving for the patient. Therefore, incrPD is a safe modality to begin dialysis and should be offered to most patients with significant RRF (3–6 ml/min/1.73 m^2^ BSA) at the start of dialysis. In order to obtain good compliance, patients should be given adequate information about the future need to increase the dose of dialysis when RRF declines. A longer preservation of RRF could be a further positive effect of this modality and favor its choice among patients on the waiting list for renal transplantation.

Two things are necessary to start dialysis with incrPD: (1) a well-organized pre-dialysis outpatient clinic able to postpone the start of dialysis [26] as well as to “build” an informed and compliant patient; (2) a close clinical and laboratory follow-up to avoid that a sudden reduction in RRF could precipitate the patient towards a condition of under-dialysis. Moreover, last but not least, some patients could receive a kidney transplant while on incrPD before switching to full-dose PD. Finally, one cannot exclude than incrPD could favor the diffusion of PD, a dialysis modality that is cost-saving in comparison to HD.
